# Immune Tolerance-Adjusted Personalized Immunogenicity Prediction for Pompe Disease

**DOI:** 10.3389/fimmu.2021.636731

**Published:** 2021-06-16

**Authors:** Anne S. De Groot, Ankit K. Desai, Sandra Lelias, S. M. Shahjahan Miah, Frances E. Terry, Sundos Khan, Cindy Li, John S. Yi, Matt Ardito, William D. Martin, Priya S. Kishnani

**Affiliations:** ^1^ EpiVax, Inc., Providence, RI, United States; ^2^ Center for Vaccines and Immunology, College of Veterinary Medicine, University of Georgia, Athens, GA, United States; ^3^ Division of Medical Genetics, Department of Pediatrics, Duke University Medical Center, Durham, NC, United States; ^4^ Department of Surgery, Duke University Medical Center, Durham, NC, United States

**Keywords:** Pompe Disease (glycogen storage disease type II), enzyme replacement therapy (ERT), immune tolerance induction (ITI), anti-drug antibodies (ADA), acid alpha-glucosidase (GAA), cross-reactive immunological material (CRIM), Tregitope, personalized immunogenicity assessment (PIMA)

## Abstract

Infantile-onset Pompe disease (IOPD) is a glycogen storage disease caused by a deficiency of acid alpha-glucosidase (GAA). Treatment with recombinant human GAA (rhGAA, alglucosidase alfa) enzyme replacement therapy (ERT) significantly improves clinical outcomes; however, many IOPD children treated with rhGAA develop anti-drug antibodies (ADA) that render the therapy ineffective. Antibodies to rhGAA are driven by T cell responses to sequences in rhGAA that differ from the individuals’ native *GAA* (nGAA). The goal of this study was to develop a tool for personalized immunogenicity risk assessment (PIMA) that quantifies T cell epitopes that differ between nGAA and rhGAA using information about an individual’s native GAA gene and their HLA DR haplotype, and to use this information to predict the risk of developing ADA. Four versions of PIMA have been developed. They use EpiMatrix, a computational tool for T cell epitope identification, combined with an HLA-restricted epitope-specific scoring feature (iTEM), to assess ADA risk. One version of PIMA also integrates JanusMatrix, a Treg epitope prediction tool to identify putative immunomodulatory (regulatory) T cell epitopes in self-proteins. Using the JanusMatrix-adjusted version of PIMA in a logistic regression model with data from 48 cross-reactive immunological material (CRIM)-positive IOPD subjects, those with scores greater than 10 were 4-fold more likely to develop ADA (p<0.03) than those that had scores less than 10. We also confirmed the hypothesis that some GAA epitopes are immunomodulatory. Twenty-one epitopes were tested, of which four were determined to have an immunomodulatory effect on T effector response *in vitro*. The implementation of PIMA V3J on a secure-access website would allow clinicians to input the individual HLA DR haplotype of their IOPD patient and the GAA pathogenic variants associated with each GAA allele to calculate the patient’s relative risk of developing ADA, enhancing clinical decision-making prior to initiating treatment with ERT. A better understanding of immunogenicity risk will allow the implementation of targeted immunomodulatory approaches in ERT-naïve settings, especially in CRIM-positive patients, which may in turn improve the overall clinical outcomes by minimizing the development of ADA. The PIMA approach may also be useful for other types of enzyme or factor replacement therapies.

## Introduction

Infantile-onset Pompe Disease (IOPD) is a fatal autosomal recessive glycogen storage disorder caused by a deficiency of the enzyme acid alpha-glucosidase (GAA), which breaks down lysosomal glycogen. The deficiency of lysosomal GAA leads to the accumulation of glycogen and damage to skeletal, cardiac and smooth muscles (1). Children who are born with IOPD present with hypotonia and hypertrophic cardiomyopathy within the first days to weeks of life, and lethal cardiorespiratory failure occurs if treatment is not initiated within the first 6 months (2). The introduction of enzyme replacement therapy (ERT) with recombinant human acid alpha-glucosidase (rhGAA) has vastly improved IOPD patient survival and quality of life. However, children who have IOPD and are treated with rhGAA can develop IgG anti-drug antibodies (ADA) to ERT. The development of high and sustained antibody titers (HSAT) results in reduced efficacy of the replacement therapy, and clinical decline (3).

The development of ADA to rhGAA is influenced by the presence or absence of endogenous GAA, defined as cross-reactive immunologic material (CRIM). Individuals who are CRIM-negative have a complete absence of GAA and are at the highest risk of ADA, whereas those who are CRIM-positive may be more immune tolerant to ERT, due to prior exposure to endogenous GAA (4). However, one-third of CRIM-positive IOPD children still develop high and sustained or intermediate ADA titers, putting them at risk for clinical decline similar to CRIM-negative individuals (5). All CRIM-negative patients develop HSAT. An Immune Tolerance Induction (ITI) protocol using a short course of Rituximab, Methotrexate, and IVIG has been successful for the treatment and prevention of ADA, and is the standard of care for all CRIM-negative IOPD children (6–8). As only one-third of CRIM-positive IOPD develop ADA, and it is difficult to predict exactly which CRIM-positive children are at high risk, the cost-benefit profile of ITI treatment with rituximab, methotrexate, and IVIG is not favorable, for these individuals. Improved methods for differentiating high-risk from low-risk CRIM-positive subjects and correctly identifying those that should be treated with ITI *versus* those who can be carefully watched instead, are needed.

**Figure 1:**
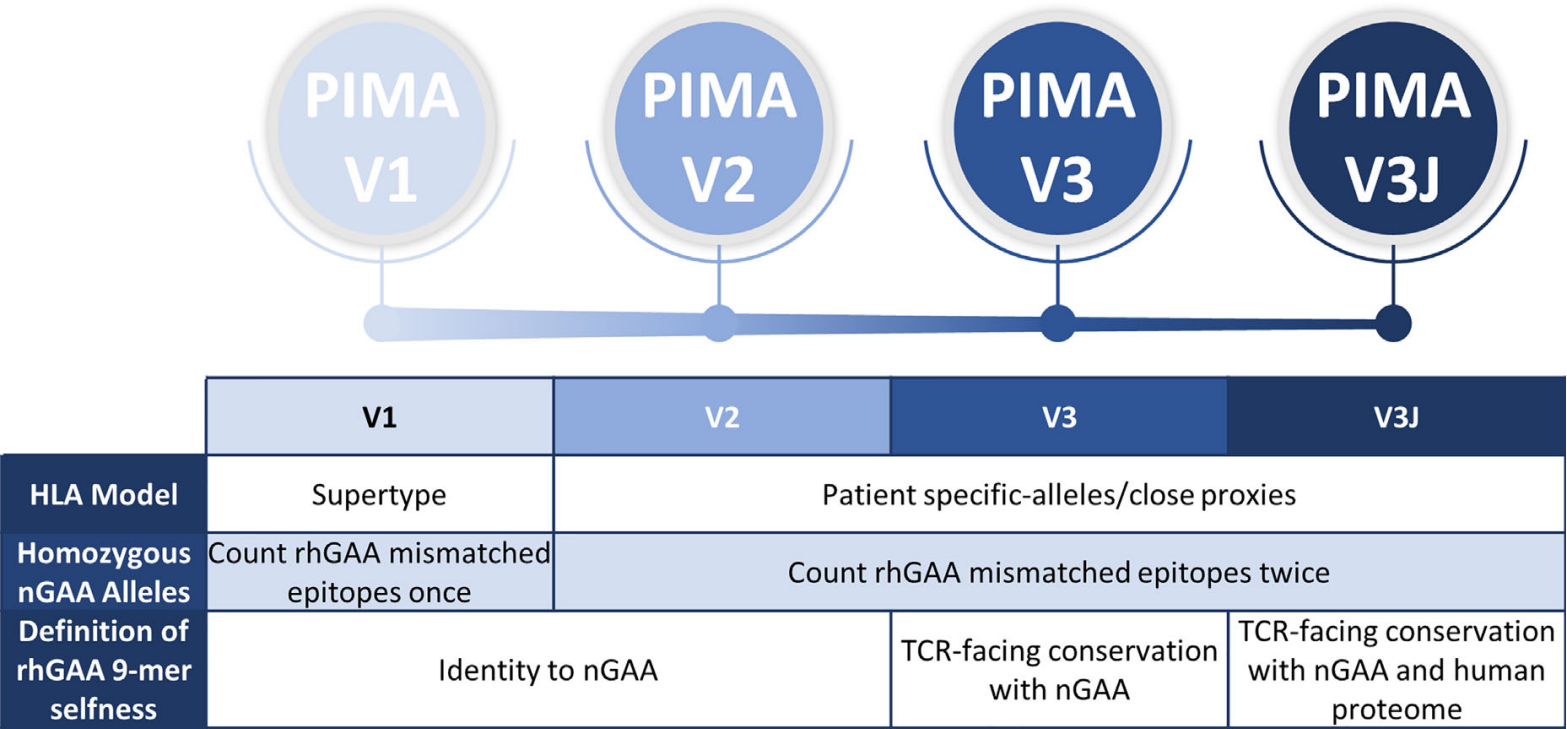
Overview and evolution of the PIMA scoring algorithms used to calculate the ADA risk assessment score. V1 was previously published. V2-V3 are intermediate steps to developing V3J as described here. V2 added subject-specific HLA DR epitope prediction, V3 added conservation with nGAA at the TCR face of epitopes, and V3J examine the potential for certain nGAA epitopes to be tolerogenic by comparing the sequence to other human genome epitopes. The most accurate for this cohort was V3J, which adjusted the prediction for T cell epitopes that are cross-conserved with other self-epitopes (not confined to nGAA). In contrast to V1, where epitopes mismatched between rhGAA and nGAA of individuals with homozygous nGAA mutations were counted once, V2-V3J included mismatched epitopes twice in the calculations, once for each allele. We then identified specific T cell epitopes in GAA that generated tolerance *in vitro*. Individual immune tolerance to nGAA sequences may diminish the risk of ERT-related ADA.

We previously established a tool for personalized immunogenicity risk assessment (now called PIMA) that quantifies T cell epitopes that differ between nGAA and rhGAA (9) using information about an individual subject’s GAA gene and HLA DR haplotype. Here we improved on the previous version of the PIMA method by taking into consideration potential tolerizing epitopes in GAA. First, we re-evaluated the original method using information from 48 CRIM-positive IOPD subjects, whose HLA and GAA genotype data were available, and we then tested progressive improvements in three new versions of the PIMA tool (V2, V3, and V3J) that weighted selected factors, such as conservation of T cell epitopes with proteins in the human genome beyond conservation with GAA (**Figure 1**). We then asked which of four versions of PIMA would best align with clinical outcomes as measured by ADA titers.

Of the new PIMA prediction models, the final version (V3J), which included more precise definition of HLA DR alleles for each subject and a correction for T cell epitopes in nGAA that may be inducing tolerance to the recombinant replacement enzyme (rhGAA), performed better than the intermediate versions. This version of PIMA integrates information generated by JanusMatrix, a computational tool that identifies T cell epitopes that have extensive conservation in the human genome (at their TCR face), which may be epitopes that activate circulating regulatory T cells (10).

We also investigated the potential for selected epitopes identified by JanusMatrix to induce regulatory T cell responses *in vitro*. Several of the putative Treg epitopes identified in GAA significantly suppress effector memory T cell response in a standardized Treg bystander assay. This important discovery of potential regulatory T cell epitopes in nGAA may improve the assessment of immunogenicity risk for IOPD and for a range of enzyme replacement therapies.

And finally, a first-generation PIMA website (Pompe-PIMA) has been developed for use in clinical decision making. Once the clinician inputs the patient-specific nGAA sequence(s) and HLA DRB1 alleles, an ADA risk estimate that is based on V3J can be calculated. After further validation and regulatory approval, the website may be used by clinicians to assess the relative risk associated with ERT therapy for their individual CRIM-positive IOPD patient.

## Materials And Methods

### Enrollment of IOPD Cohorts

#### Recruitment

Children with a confirmed diagnosis of IOPD were enrolled in Duke University Medical Centers. IOPD was defined as the presence of hypertrophic cardiomyopathy in the first year of life. Parents of subjects were provided with a written consent approved by the Institutional Review Board (IRB) [(Pro00001562; Determination of Cross-Reactive Immunological Material (CRIM) Status and Longitudinal Follow-up of Individuals with Pompe disease; LDN6709 Site 206; ClinicalTrials.gov NCT01665326]. Subjects were selected for the present study based on the following inclusion criteria: 1) a confirmed diagnosis of Infantile-onset Pompe disease (IOPD), 2) CRIM-positive status determined as described previously (4), 3) received ERT with rhGAA, 4) had a skin/blood sample available for HLA haplotyping, 5) did not receive immune tolerance induction, and 6) availability of at least 6 months of follow-up data. Clinical data including CRIM status, *GAA* variants, GAA enzyme activity, age at ERT initiation, and longitudinal anti-rhGAA IgG antibody titers were extracted from medical records (**Supplementary Table S1**).

#### CRIM Status, HLA Typing and *GAA* Sequencing

CRIM status was assessed by Western blot reactivity to a pool of monoclonal and polyclonal anti-GAA antibodies capable of recognizing both native and recombinant GAA (11, 12) from subject’s fibroblast cultures and/or PBMC (4, 13). Study subject HLA DR haplotypes were determined by PCR, using a sequence-specific oligonucleotide probe (SSP) typing test (One Lambda, Inc.). Mutations in the nGAA gene were determined by PCR amplification followed by Sanger DNA sequencing. The methodology employed here was developed by the Duke University Health System Clinical Molecular Diagnostic Laboratory (4).

#### ADA Titers and Classification of Subjects

ADA titers were determined by Sanofi Genzyme using enzyme-linked immunosorbent assays (ELISA) and confirmed using radioimmunoprecipitation as described previously (6). Subjects whose ADA titers repeatedly exceed 51,200 after more than 6-months on rhGAA were classified as high and sustained antibody titers (HSAT) (5, 11). Subjects whose ADA titers fell between 12,800 and 51,200 within the first year of ERT were classified as sustained intermediate titers (SIT). Based on ADA titers, subjects were stratified into two groups; 1) High ADA, subjects who developed ADA titers in HSAT or SIT range (≥12,800) and 2) Low ADA, subjects who maintained ADA titers of ≤6,400.

### Immunoinformatic Assessment of GAA: The Evolution of PIMA and Selection of T Cell Epitopes

The PIMA approach to assessing an individual patient’s HLA-specific risk for immunogenicity has been described previously (9). Each patient’s nGAA sequence, as well as the reference sequence for rhGAA, is parsed into overlapping 9-mer frames by an epitope prediction tool called EpiMatrix (14, 15) and each frame is evaluated with respect to the specific Class II HLA DR alleles expressed by the patient. The EpiMatrix algorithm is based on coefficient matrices representing all 20 natural amino acids and nine peptide binding pockets for each HLA allele, so that the coefficients for each amino acid in a novel peptide can be summed and normalized to generate a Z-score indicative of binding likelihood. The coefficients are derived based on empirical binding data, and Z-scores above 1.64 (the top 5% of a distribution of random peptides) are considered significant hits, likely to bind. Each 9-mer frame is assigned a normalized z-score using EpiMatrix; this z-score is used in the calculations.

**Figure 2:**
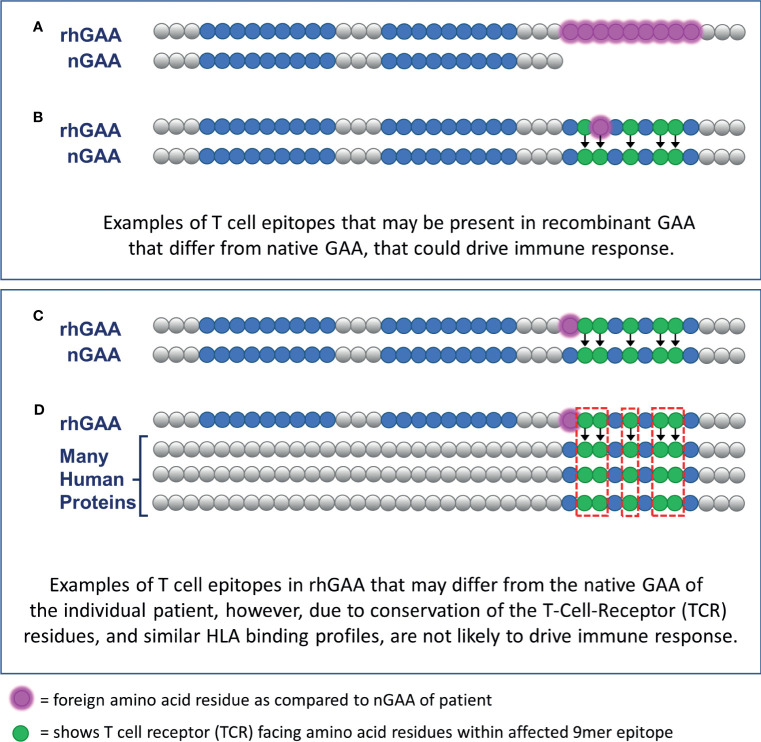
Epitope differences in therapeutic GAA from endogenously expressed native GAA predicted to drive ADA or be tolerized. CRIM-positive IOPD subjects who express residual nGAA may be tolerant to epitopes conserved, for their HLA, with the rhGAA replacement protein. **(A)** T cell epitopes contained within the rhGAA may be recognized as “foreign” if they are within the truncated or mutated portions of the patient-specific nGAA. **(B)** T cell epitopes within the rhGAA that contain T cell receptor (TCR)-facing residues that are different from those found in nGAA may be sufficient to generate a different T cell phenotype response. **(C)** T cell epitopes within the rhGAA that contains different MHC-facing residues but the same TCR-facing residues as epitopes found in nGAA are predicted to be tolerated by the immune system (this hypothesis was included in PIMA V3). **(D)** The presence of a T cell epitope in the rhGAA sequence with TCR-facing residues highly cross-conserved with several self-human proteins may not appear as foreign and would also be tolerated by the immune system. (This hypothesis was included in PIMA V3J).

EpiMatrix focuses on HLA DR, as it is usually expressed at the highest levels on antigen presenting cells (16, 17) and has been associated with therapeutic protein immunogenicity; no predictions were performed on HLA DP or DQ. Those 9-mer peptides predicted to bind to HLA DR, found in the rhGAA sequence, but absent in at least one of the patient’s nGAA alleles, are considered potential inflammatory (T effector) epitopes and included in the calculation of the PIMA score. Henceforward, this approach will be referred to as PIMA V1. **Figure 2** illustrates how new epitopes introduced from the rhGAA therapeutic could potentially drive an ADA response in IOPD subjects. (**Figures 2A, B**). Notably, PIMA does model the impact of stop codons, where every section of rhGAA following the stop codon is considered potentially foreign to the patient, and of frame-shift mutations, where the out-of-frame translations are compared to rhGAA (and to the remainder of the human proteome in V3J) to assess the foreignness of the therapeutic protein. PIMA is not yet able to model the impact of splice-site variants due to the heterogeneous nature of gene products from these mutations.

In this study, three additional candidate scoring approaches were evaluated: PIMA V2, V3 and V3J. For PIMA V1, predictive models for the following supertype alleles were available: HLA DRB1*0101, *0301, *0401, *0701, *0801, *1101, *1301 and *1501. Since the publication of V1, many new HLA DR allele epitope prediction models have been developed for use in EpiMatrix. PIMA V2 evaluated whether expanding the set of HLA DR alleles used for T cell epitope prediction improved the performance of the tool. In V2, IOPD subject-specific subtypes were used instead of the eight supertypes (e.g. HLA DRB1 subtype 0403 instead of DRB1 supertype 0401). If a prediction model for a patient’s HLA DR allele is not available in our EpiMatrix tool, a close proxy was selected based on HLA DR binding pocket similarity (by examining the preferred side chains) and that was used for T cell epitope prediction. The new, more precise subject-specific subtypes were also incorporated into the subsequent V3 and V3J analyses.

The next step was to refine the selection of T effector epitopes for the third PIMA model (V3). In previous models, we considered all epitopes that were not identical in sequence to nGAA to be potential T cell epitopes. We re-assessed these potential T effector epitopes in V3. Instead of automatically counting non-identical sequences as T cell epitopes, we considered only whether the epitopes were different from nGAA at the T cell receptor face. If so, they counted as T effector epitopes, but if they were conserved at the TCR face (even if their HLA DRB1 HLA-face was different, and if it was still predicted to bind to the same HLA DRB1), we considered the epitopes to be ‘null’ or not T effector epitopes (**Figure 2C**). To perform this analysis, we used the JanusMatrix tool (10). In retrospective and prospective studies we have determined that TCR conserved epitopes may be tolerated, deleted during the thymic selection process, or actively regulatory (18–20). For PIMA V3, GAA-like epitopes were excluded from the calculation of the PIMA score.

In some subjects, mutations are caused by frameshifts. For each frameshift and non-sense mutations, the anticipated expressed GAA protein product is compared to the therapeutic rhGAA sequence. The sequence of the rhGAA which does not align with the truncated protein product is considered to be mismatched and is scored as a foreign protein as follows: The mismatched sequence is parsed into 9-mer frames and evaluated for potential HLA binding hits to the patient’s specific HLA DRB1 haplotype. For PIMA V1 and PIMA V2, the HLA-epitope hit values are then added up to calculate to overall PIMA score. In the case of V3 and V3J, the predicted binding epitopes are further evaluated for T cell receptor facing residues and cross-conservation to the human proteome (V3J).

Beyond finding epitopes that may be tolerated, JanusMatrix can be used to identify putative regulatory T cell epitopes if the TCR face is extensively conserved with other epitopes from the human genome (10, 21, 22). Therefore we searched for putative Treg epitopes in GAA using the study subject HLA DR alleles, and then identified potential Treg epitopes, specific to each subject, for PIMA V3J. This version of PIMA discounts additional epitopes defined by JanusMatrix that may or may not be conserved in nGAA but are conserved (at their TCR face) within *other* human proteins (**Figure 2D**). We used the UniProt Reviewed Human Proteome as the database for comparison (23, 24). T cell epitopes that had high JanusMatrix scores, indicating high conservation to other human proteins, were not included in the calculation of the PIMA score. Some of these ‘regulatory’ epitope sequences were produced as peptides and were also evaluated *in vitro*.

### Selection and *In Vitro* Validation of Putative Regulatory T Cell Epitopes in GAA

The first step in the search for Treg epitopes was to use EpiMatrix to analyze the sequence of recombinant GAA replacement enzyme. This analysis considered the complete GAA sequence and the globally representative set of HLA DR supertype alleles (25). Several categories of putative T cell epitopes were identified based on their EpiMatrix cluster score and their ability to bind across multiple HLA DRB1 alleles. Next, to determine cross-conservation with the human proteome, each of the clusters was screened using JanusMatrix. In general, JanusMatrix Human Homology Scores above two are considered significant, indicating an elevated level of conservation between the TCR-facing features of the input peptide and the TCR-facing features of the proteins resident within the human genome. Those epitopes with higher conservation scores were considered to be putative Treg or regulatory T cell epitopes. **Supplemental Table S2** describes the peptides that were tested, including their predicted binding affinity (using EpiMatrix) and their corresponding JanusMatrix score. T effector epitopes that were used as controls are also shown in this table.

Twenty-one GAA-derived putative regulatory T cell epitopes were identified and synthesized for *in vitro* evaluation and validation studies. Twelve were promiscuous epitopes that were predicted to bind across multiple HLA DRB1 alleles (and therefore relevant to a wide range of haplotypes), while nine putative GAA regulatory T cell epitopes were more HLA DR restricted by the HLA DRB1 of the individual IOPD subjects included in the study cohort. T cell assays were performed using a diverse panel of healthy donor PBMCs (as subject-specific PBMCs were limited for *in vitro* studies). The positive (regulatory T cell epitope) control for this assay was a Treg epitope similar to the previously identified Tregitopes (26), FV621. This control peptide is a Factor V peptide that modulates CD4+ memory T cell responses and induces bystander suppression of T effector immune response *in vitro* in a standardized Tetanus Toxin Bystander Suppression Assay (TTBSA) (27).

### HLA Binding Assays

The major histocompatibility complex proteins (MHC, also known as HLA in humans) play a critical role in the development of an effective immune response or in activating both effector and regulatory T cells to induce, or diminish immune responses, respectively. The twenty-one putative regulatory T cell epitopes from GAA were tested for *in vitro* binding to HLA DRB1*0101, *0301, *0401, *0701, *1101, *1301 and *1501 alleles. The HLA DRB1 alleles selected for the HLA binding assays represent families of Class II HLA DRB1 alleles that share similar binding peptide side-chain preferences for their binding pockets (25).

The HLA binding assay used at EpiVax was originally described by Steere et al. (28), has been standardized for in-house validation of *in silico* binding predictions. This binding assay has been described in detail in previous publications (28–30). A seven-point binding assay (0.01, 0.1, 0.3, 3.0, 10.0, 30.0 and 100.0 μM) is performed for each test peptide, in triplicate. The HLA binding information is used to calculate the IC_50_, or the concentration at which the peptide inhibits 50% of the labeled control peptide’s specific binding.

### Human Peripheral Blood Mononuclear Cells (PBMCs)

PBMCs from healthy donors were isolated from leukocyte reduction filters purchased from the Rhode Island Blood Center (RIBC) in Providence, RI. High-resolution HLA Class II DRB1 haplotyping of donor PBMCs was performed at the Transplant Immunology Laboratory at Hartford Hospital in Hartford, CT. Donors’ age and sex are provided however race, ethnicity, and medical history are not available due to the anonymous nature of the blood donation process.

All assays were performed in RPMI complete medium: RPMI-1640 + GlutaMax (Life Technologies) containing 10mM HEPES buffer (Life Technologies), 2mM L-glutamine (Life Technologies), 50µg/ml Gentamicin (Life Technologies), 10% Human AB serum (Sigma), MEM Non-essential amino acids (Gibco) and 55µM β-Mercaptoethanol (Gibco).

### Tetanus Toxoid Bystander Suppression Assay (TTBSA)

The TTBSA measures the inhibitory capacity of potential regulatory peptides on the recall response of human CD4 T cells to the tetanus toxoid (TT) antigen was adapted for validation of Treg epitopes and previously described (27, 31). TT vaccination is a routine, nearly universal immunization, resulting in memory T cell responses that persist for many years (32). Therefore PBMCs are considered to be a reliable source for *in vitro* assays that require TT-specific memory T cells.

Briefly, PBMCs were labeled with CFSE cell proliferation dye (eBioscience) and rested overnight at 37°C, 5% CO_2_. The following day cells were stimulated with Tetanus toxoid (TT) (Astarte Biologics, cat no. 1002) at 0.5 μg/ml alone and in combination with the putative regulatory peptides or control peptide at 8, 16 or 24 μg/ml, then incubated for 6 days and analyzed by flow cytometry on day 7. CD4+ T cell proliferation, T effector activation and the ratio of regulatory to effector T cells were measured.

### Statistical Analysis

Association between predictors of ADA response and outcome were evaluated by Chi-square test, or Fisher’s Exact test in the case of small sample sizes, using GraphPad online tools (GraphPad Software). Prediction metrics (sensitivity, specificity, positive predictive value, negative predictive value, odds ratio) were evaluated using Microsoft Excel (2016) (33). Statistical analysis was performed using Prism software (GraphPad version 8.3). The Student’s *t*-test (unless otherwise indicated, unpaired, two-tailed) was used to compare the significance of differences between TT stimulated cells to Tregitope treated cells or the indicated experimental groups. Differences were considered significant when *p* < 0.05 (*), very significant when *p* < 0.01 (**), highly significant when *p* < 0.0002 (***), and extremely significant when *p* < 0.0001 (****).

## Results

### GAA PIMA Scores as a Predictor of ADA Status

HLA and GAA genotype data were collected from 62 individuals with IOPD in an international cohort of IOPD subjects enrolled in the Duke University IRB-approved study. These individuals include 19 CRIM-positive participants analyzed as part of our published (V1) pilot study (9). Fourteen of the 62 subjects were excluded from this analysis due the presence of splice site mutations resulting in indeterminate protein products (6) that is characteristic of late-onset Pompe disease (8). At this phase of the PIMA development splice-site variants have not been integrated into the analysis due to the heterogeneous nature of gene products from these splice-site mutations. Among the remaining 48 IOPD subjects, 19 (40%) developed high ADA titers and 29 (60%) developed low ADA titers.

**Figure 3:**
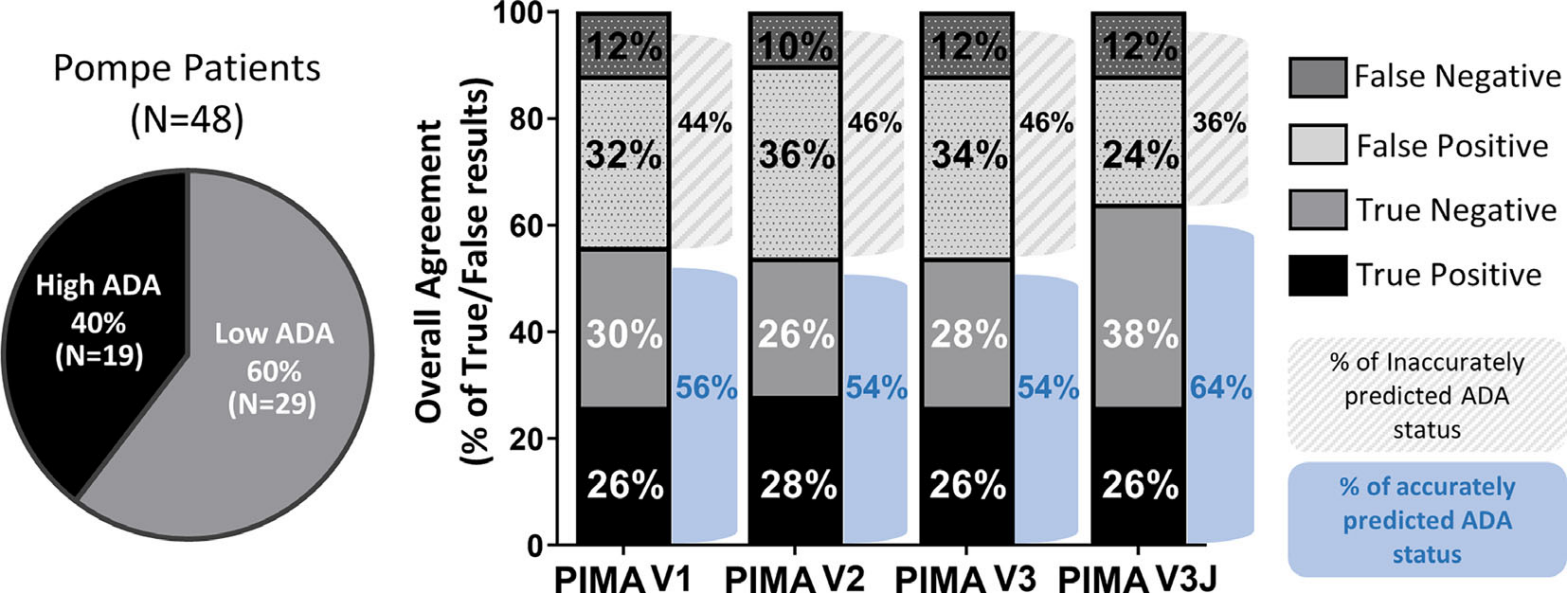
Overall agreement of the four PIMA scoring algorithms as predictors of ADA status. Among the 48 IOPD subjects evaluated, 19 (40%) developed high ADA titers; this includes subjects with high and sustained antibody titers (HSAT) as well as sustained intermediate titers (SIT). The remaining 29 subjects (60%) developed low ADA titers. PIMA V1 (previously published) accurately predicted the ADA status for 56% of the IOPD subjects. Versions V2 and V3 accurately predicted 54% of subjects, thus further adjustment was explored. Adjusting for potential Treg epitopes with V3J improved accuracy to 64% of the subjects.

Using a score threshold of +10, PIMA V1 correctly predicted ADA status for 27 (56%) of 48 subjects, the intermediate versions V2 and V3 correctly predicted for 54% of the subjects and PIMA V3J correctly predicted for 64% of the subjects (**Figure 3**). For each of the analyses, correct predictions included both True Positive (predicted to and did develop high ADA) and True Negative (predicted not to and did not develop high ADA) predictions. False Negative predictions (subjects who developed high ADA contrary to predictions of low ADA) represented the smallest set among all predictions at 10-12% (5-6 out of 48 subjects).

### Regression Model Improves Prediction Outcome

The four PIMA scoring algorithms were further evaluated as predictors of high ADA development in IOPD using univariate and multivariable logistic regression analysis. Odds ratios (OR) and 95% confidence intervals (95% CI) for the development of high ADA titers were calculated. Two-sided P-values <0.05 were employed to assess significance. In the univariate logistic regression analysis, IOPD subjects with PIMA scores above 10 had increased odds of developing high ADA titers compared to subjects with scores below 10.

As shown in **Table 1**, PIMA V3J reported the highest OR of 4.12 (95% CI 1.24-15.01), which was statistically significant. The regression model was slightly improved by incorporating the age of the subject at initiation of ERT as a covariable, as both PIMA score >10 and age were significantly associated with the high ADA outcome. After adjusting for age at ERT initiation, PIMA V3J scores of greater than 10 were associated with the highest adjusted OR of 4.40 (95% CI 1.21-18.21), also statistically significant (**Table 1**). For the combined risk model, age at ERT initiation was divided into two categories using the mean (19 weeks) as a cutoff (>19 and <19 weeks).

**Table 1:** PIMA V3J and age at ERT initiation are significant predictors of high ADA in univariate and multivariable logistic regression analyses.

↓version	UNIVARIATE LOGISTIC REGRESSION		MULTIVARIABLE LOGISTIC REGRESSION (age in Weeks at ERT as a covariable)			
	PIMA OR (95% CI)	p-val	PIMA OR (95% CI)	p-val	Age OR (95% CI)	p-val
**PIMA V1**	2.32 (0.71-8.21)	0.1728	3.45 (0.93-15.04)	0.0770	1.07 (1.019-1.133)	0.0105*
**PIMA V2**	2.27 (0.67-8.58)	0.1997	3.40 (0.87-16.23)	0.0945	1.07 (1.019-1.132)	0.0110*
**PIMA V3**	2.02 (0.62-7.14)	0.2544	2.74 (0.75-11.54)	0.1418	1.07 (1.017-1.127)	0.0123*
**PIMA V3J**	4.12 (1.24-15.01)	0.0246*	4.40 (1.21-18.21)	0.0296*	1.06 (1.012-1.122)	0.0214*

Odds of high ADA were 2.32-4.12 times greater for subjects with PIMA V1-V3J scores >10 according to univariate logistic regression models (left). The odds ratio for the PIMA V3J model was statistically significant (p=0.0246). Multivariable logistic regression models incorporating both PIMA V1-V3J and age in weeks at initiation of ERT (right) indicate that increasing age consistently conferred 6-7% increased odds of high ADA per week after adjusting for PIMA score. In the PIMA V3J model, both the PIMA score and age variables were statistically significant (p=0.0296 and p=0.0214, respectively). Notably, the inverse interpretation is also true. For example, in the V3J univariate model, subjects with PIMA scores <10 were 4.12 times more likely to maintain low ADA. In the V3J multivariable model accounting for both age and PIMA score, patients with scores <10 were 4.4 times more likely to maintain low ADA, while each week of increasing age conferred 6-7% lower odds of maintaining low ADA. *Significant, two-sided p-value < 0.05.

Considered together, subjects with PIMA V3J scores >10 and initiation of ERT after 19 weeks of age were 8.23 times more likely to develop high ADA than all other subjects (**Table 2**). Viewing the data from a clinical importance perspective (using PIMA to identify subjects at low risk), subjects with PIMA V3J scores <10 and ERT initiation prior to 19 weeks were 12.7 times more likely to have low ADA compared to all other subjects. If validated in future studies, the V3J PIMA score may be clinically useful for identifying IOPD subjects who may not need to be treated with ITI. We have also compared the area under the ROC curve (AUC) among univariate and multivariable logistic regression models which confirm the improved accuracy of V3J over V1 and intermediate versions V2 and V3 (**Supplementary Figure S1**). A multivariable regression using both PIMA and age as independent variables to predict high ADA titer status indicated that regression coefficients for both factors were significant, and the joint threshold model (PIMA >10 and age@ERT >19 wks) had the lowest p-value and highest AUC of all the models we explored.

**Table 2:** Combined logistic regression model for high and low ADA risk.

OUTCOME	Predictor	Univariate Logistic Regression	
		OR (95% CI)	p-val
**HIGH ADA**	**PIMA V3J>10 & AGE @ ERT >19 WKS**	8.23 (2.28-34.31)	0.00206**
**LOW ADA**	**PIMA V3J<10 & AGE @ ERT <19 WKS**	12.7 (2.15-244.25)	0.0202*

Combined logistic regression models for ADA risk indicate that IOPD subjects with PIMA V3J scores >10 and ERT initiation after age 19 weeks were 8.23 times more likely to develop high ADA than all other subjects, while subjects with PIMA V3J scores <10 and ERT initiation prior to 19 weeks were 12.7 times more likely to maintain low ADA compared to all other subjects. *Significant, two-sided p-value < 0.05, **Highly significant, two-sided p-value < 0.01.

### Selection of GAA-Tregulatory Peptides and HLA Binding

**Figure 4:**
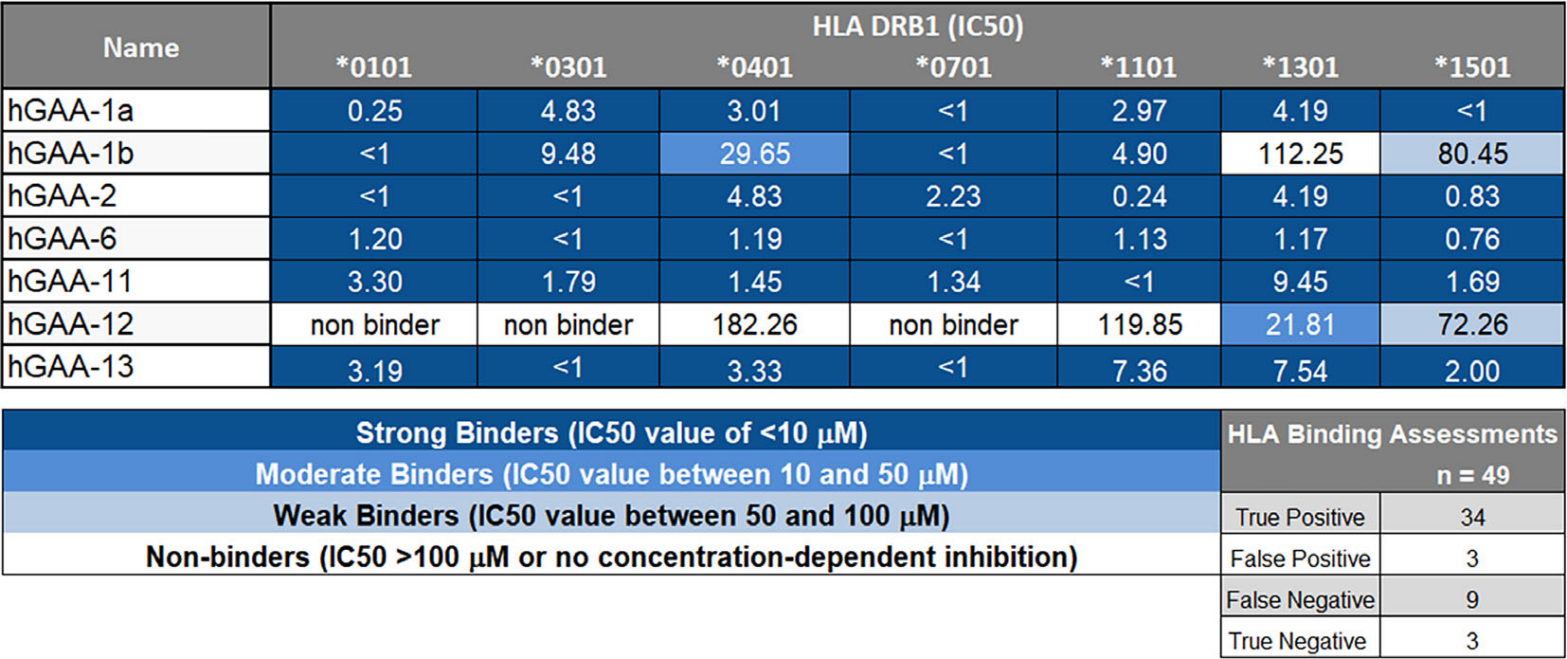
GAA-peptides bind to HLA DR1 as predicted. Selected GAA peptides were evaluated for HLA DRB1 binding *in vitro* and IC_50_ values were calculated. hGAA-1a, hGAA-1b, hGAA-2, hGAA-6, hGAA-11 and hGAA-13 FV621 peptides bound with the multiple alleles tested (DRB1*0101, *0301, *0401, *0701, *1101, *1301 and *1501) whereas hGAA-12 was predicted to be more HLA-restricted and consequently had limited binding to HLA. A seven-point competition assay using a validated control peptide was performed; color coding reflects binding affinity IC_50_ was determined by interpolation. Using a standard Z-score threshold of 1.64 (top 5%), overall positive predictive value for EpiMatrix predictions was 92%, with sensitivity of 79%. False negatives are not uncommon when testing peptides containing significant predictions for several alleles (EpiBars), as many contain “near-miss” Z-scores in the top 10% of predicted peptides. Note that peptide GAA-12 and 13 were not selected for promiscuity: they were designed for individualized testing in a specific patient for which both the mutation and the HLA DRB1 allele restriction concurred.

To investigate the hypothesis that there may be tolerogenic peptides in GAA, we used JanusMatrix to identify putative tolerogenic epitopes. Epitopes that were predicted to bind promiscuously across multiple human class II HLA-DRB1 molecules were selected for testing *in vitro*, as were other epitopes that were more restricted by HLA DRB1 class. To confirm HLA binding before testing *in vitro* bystander T cell assay, we performed 7-point HLA DR binding assays. See **Supplementary Table S1** for list of all peptides tested in the *in vitro*. Within twenty-one tested peptides, we found that the 12 GAA peptides showed moderate to strong binding to the panel of multiple HLA alleles, whereas others were somewhat more restricted in the breadth of binding to the full range of HLA DR alleles (data not shown). **Figure 4** summarizes the HLA-binding results for the putative Treg epitopes in GAA evaluated in TTBSA.

### Immunomodulatory Effect of GAA-Derived Peptides on the Tetanus Toxoid Mediated Recall Response of CD4 T Cell Proliferation

**Figure 5:**
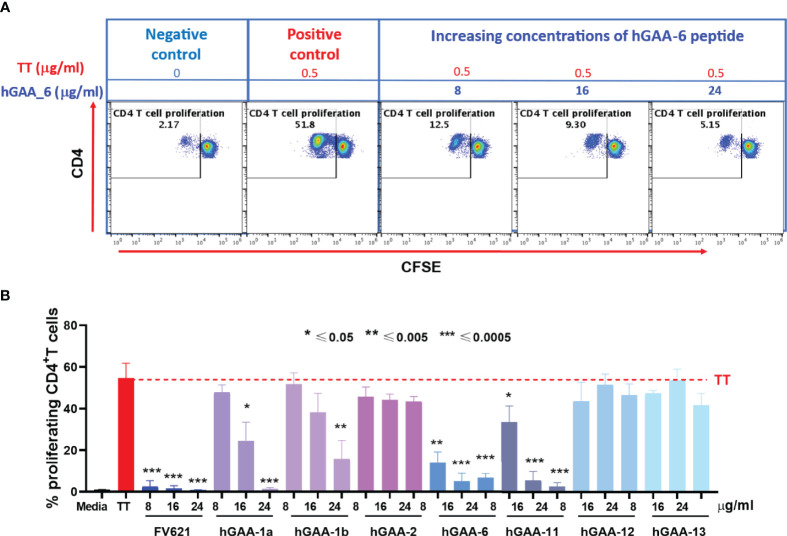
GAA-derived peptides inhibit memory CD4 T cell response to Tetanus Toxoid (TT) in healthy donors. **(A)** Representative flow cytometry dot plots show CD4 memory T cell proliferative response to TT and dose-dependent inhibition by hGAA-6 peptide. **(B)** Inhibition of CD4+T cell recall response by GAA-peptides in TTBSA. PBMCs from healthy donors were stimulated with 0.5 µg/ml of TT with or without FV621 or GAA-peptides and analyzed at six days post-stimulation by flow cytometry for inhibition of CD4+ T cell proliferation. Data are the representative donor from 5 donors in the experiments. Significant suppressive capacity of CD4+ T cell proliferation was observed for 4 putative Treg peptides in GAA confirming their regulatory potential across all donors tested. P values * ≤ 0.05, ** ≤ 0.005 and *** ≤ 0.0005 represents statistical significance between peptide stimulation *vs* TT using a two-tailed t test. GAA peptides 1a, 1b, 6 and 11 significantly suppressed the expansion of TT-memory T cells in this *in vitro* assay.

To determine the magnitude of immune tolerance induced by the 21 pre-selected putative Treg epitopes and 2 IOPD patient-specific Teff epitopes (**Supplementary Table S2**), we performed a Tetanus Toxoid Bystander Suppression Assay (TTBSA) for Treg epitopes and measured their capacity to inhibit the proliferative response to TT in PBMCs derived from a panel of six healthy donors and selected 7 peptides with potential inhibitory capacity compared to validated positive control FV621 (data not shown). These 7 peptides were re-evaluated in an additional panel of 5 healthy donors for the inhibition of TT-induced memory response (**Figure 5**). Four of the 21 peptides (hGAA-1a, hGAA-1b, hGAA-6, hGAA-11) significantly inhibited memory CD4+ T cell proliferation across all the donors tested. We have also tested 2 Teff epitopes in TTBSA in healthy donor PBMCs and found that they were not inhibitory (**Supplementary Figure S2**).

As shown for one representative donor in **Figure 5A**, Tetanus Toxoid stimulation usually expanded CD4+ T cell proliferation by ten to twenty-five-fold in a CFSE dilution assay. The addition of one of the 21 GAA-peptides (hGAA peptide 6) significantly suppressed proliferation of CD4+ T cells to TT in a dose-dependent manner (75%-90%). As shown in **Figure 5B**, the FV621 T reg epitope positive control also significantly inhibits TT-induced memory CD4+ T cell proliferation. **Figure 5B** shows the effect of selected GAA peptides on the inhibition of memory CD4+ T cells. GAA peptides 1a, 1b, 6 and 11 significantly inhibited TT-induced CD4 T cell proliferation in a dose-dependent manner (observed in TTBSA for 11 donors), while none of the other peptides had the same effect.

### GAA-Derived Peptides Increased the Ratio of Tregs to Teff Cells

**Figure 6:**
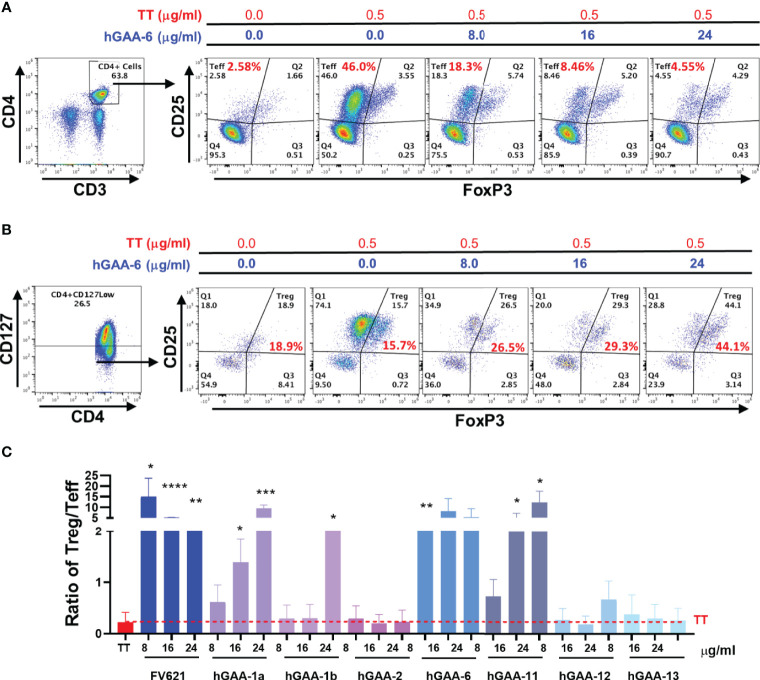
GAA-peptides modulate T regulatory to T effector cell ratio. Healthy donor PBMCs were stimulated with Tetanus Toxoid (TT) with or without GAA-selected peptides for 7 days and analyzed by flow cytometry. **(A)** Representative flow cytometry dot plots show the effect of hGAA-6 peptide on the inhibition of T effector (CD4+CD25^hi^FoxP3^int^) cells for a single donor. **(B)** The effect of hGAA-6 peptide on regulatory T cells (CD4+CD127^low^CD25^hi^FoxP3^hi^) in the representative donor is shown. **(C)** Representative histogram indicates the effect of selected GAA peptides on the Treg to Teff ratio in an individual donor. GAA peptides 1a, 1b, 6 and 11 significantly increased the Treg : Teff ratio similar to the FV621 Tregitope control. P values * ≤ 0.05, ** ≤ 0.005, *** ≤ 0.0005 and **** ≤ 0.00005 represents statistical significance between peptide stimulation at a given concentration *vs* TT using a two-tailed t test.

To further characterize the inhibitory capacity of down selected GAA derived peptides on the CD4+ T effector cell populations and investigate its impact on Tregs, we evaluated the effect of these peptides on cell surface markers in PMBC obtained from five healthy donors with diverse HLA DRB1 haplotypes. CFSE labeled PBMCs from the donors were stimulated with Tetanus Toxoid (TT) in the presence or absence of GAA-derived peptides or FV621 (as a positive control peptide) for 6 days and the proliferation of T effector and T regulatory cells was assessed. Tregs were identified by the expression of CD127^low^, CD25^hi^ and FoxP3^hi^ (FoxP3 is a transcription factor and major regulator of Treg development but is also transiently expressed in activated T effector cells) (34) while CD4+ T effector cells were identified as CD25^hi^FoxP3^int^ in the CD4+ gated population. Data from a single representative donor in **Figure 6A** shows an expansion of CD4+CD25^hi^FoxP3^int^ T effector cells in the presence of TT alone, while co-treatment of the cultures with increasing concentrations of GAA-derived peptides significantly reduced the percentage of activated CD4+ T effector cells. In parallel, we observed a dose-dependent increase in the percentage of Tregs in cultures treated with GAA-derived peptides (**Figure 6B**).

We hypothesize that the increased frequency of Tregs that is observed when PBMC cultures of activated TT-specific effector T cells are treated with increasing concentrations of GAA-peptides may be due to (1) conversion of T effectors to adaptive Tregs; (2) elimination of T effectors from the mixed population of cells due to killing by Granzyme B (35); or (3) proliferation of natural Tregs. Previous studies in D011.10 mice showed that treatment with other Treg epitopes *in vivo* converted (OVA) antigen-specific T effector cells to regulatory T cells (36). The potential for conversion of T effectors to adaptive Tregs is supported by additional published studies which show that Tregitope treatment of human PBMCs *in vitro* converted tetramer-stained Birch Pollen specific T effector cells to adaptive Tregs (26).

The ratio of activated T regulatory cells to T effector cells may be a determining factor in the maintenance of tolerance and in the potential for tolerance to ERT as well as for treatment of allergic and autoimmune diseases (37, 38). Here we have found that GAA-derived peptide treatment in the presence of TT also shifts the balance of T effector cells and T regulatory cells by increasing the ratio of Treg to Teff cells, also in a dose-dependent manner (**Figure 6C**).

## Discussion

Among the 48 IOPD subjects evaluated in this study, 19 (40%) developed high ADA titers, which included patients who exhibited sustained ADA titers of ≥12,800. Using the existing personalized immunogenicity risk assessment (PIMA) Version 1 (V1) (9), we found that ADA status was predicted accurately for 56% of subjects in the cohort. Intermediate versions 2 and 3 which used more specific HLA DR allele models (patient specific rather than supertype) and assessed for TCR conservation with GAA, respectively, were not significantly better than Version 1. Notably, in multivariable regression models, both age at initiation of ERT and PIMA score were significant indicators of the likelihood of developing high ADA to rhGAA. We believe that the significant effect size for age at ERT initiation underscores the importance of early assessment, while the significant finding for PIMA score supports the potential benefits of delaying ITI treatment for those individuals at lower risk of ADA. Across versions, 5-6 subjects developed high ADA despite having PIMA scores below 10. The specific characteristics of these subjects will be considered as the PIMA scoring algorithm is refined in future versions.

An additional version (PIMA V3J) adjusted for potential regulatory T cell epitopes using JanusMatrix was also tested. This improved version that integrated putatively tolerated and tolerogenic epitopes predicted ADA status accurately for 64% of subjects. To explore the interesting hypothesis that these epitopes might down-modulate T effector responses to ERT we used a validated *in vitro* T cell assay (The Tetanus Toxoid Bystander Suppression Assay (TTBSA) (27) to examine their potential regulatory effects, using blood from naïve donors. We confirmed that four of the 21 GAA epitopes tested *in vitro* appear to be significantly immunomodulatory. These four peptides inhibited the expansion of Tetanus Toxoid-specific memory CD4+ T cells in a standardized Treg assay (the TTBSA), similar to other well-defined Treg epitope peptides. Others have reported an absence of immune responses to some of the epitopes that have been confirmed to be regulatory here (39). The reasons for which 17 of the 21 peptides were not tolerogenic in the same assay is unknown, although epitope processing (lack of proper processing and presentation on the cell surface) may play a role.

In subjects who have circulating Tregs that recognize these epitopes in GAA, treatment with the drug may activate antigen-specific T regulatory cells, contributing to the induction of tolerance to the GAA therapy while limiting the development of ADA. In future studies, we intend to assess the effect of these peptides in the TTBSA with blood samples from IOPD subjects, while also testing additional GAA T effector and GAA Treg epitopes using naïve donor and IOPD subjects’ T cells. In keeping with the hypothesis, tolerance to GAA has been observed in late-onset Pompe Disease (LOPD (40).

The discovery of Treg epitopes in self proteins has implications for protein therapeutics and may help explain why some subjects unexpectedly develop tolerance to ERT or blood factor therapy. For example, we have identified a Treg epitope in Factor V that may induce tolerance to Factor VIII in certain hemophilia A subjects (27). The potential for human proteins to have internal Treg epitopes may transform the prediction of ADA development for Pompe patients and has important implications for other protein-based replacement therapies (41). The putative GAA Treg epitopes are similar to Treg epitopes first discovered in IgG (Tregitopes, T regulatory epitopes) (26) in 2008. These Tregitope sequences were synthesized as peptides and encoded in viral vectors (AAV) and shown to suppress inflammatory responses to co-administered antigens (Ag) (such as diabetes antigens, AAV capsid, MOG protein, OVA, and other antigens) *in vitro* and *in vivo* (36, 42–44). Co-delivery of Tregitopes in conjunction with target Ag appears to be critical to the induction of antigen-specific tolerance (45); antigen-specific tolerance may also be operational in the setting of enzyme replacement, providing the IOPD individual has circulating Tregs that recognize these GAA Treg epitopes with tolerance to rhGAA.

In the context of GAA, we hypothesize that exogenous rhGAA may be able to induce tolerance in CRIM-positive children due to the engagement of pre-existing GAA-specific regulatory T cells. Circulating Tregs may be found in subjects who have been exposed to nGAA that contains these GAA sequences. Other subjects (such as CRIM-negative subjects, or other subjects who have key mutations or truncations in the region of the GAA Treg epitope), may not have Tregs that respond to these sequences. Tolerance to GAA may be ‘personalized’ since it is both native GAA-sequence-specific and HLA DR-haplotype dependent. Therefore, a personalized immunogenicity risk assessment such as the PIMA V3J tool may be the most accurate means of assessing the risk of an immune response to replacement rhGAA.

The means by which the GAA-specific immunomodulatory T cells modulate immune responses deserves further study. Their effect may be due to i) production of immunosuppressive cytokines, e.g., TGFβ, IL-10 and IL-35, ii) upregulation of effector T cell-specific transcription factors important for the expression of CXCR3 and survival of Tregs (46)(e.g., T-bet), iii) competing with effector T cells for the growth factor IL-2 by sustained expression of the IL-2Rα subunit, CD25, iv) inducing cytolysis of T effector cells by producing perforin and granzyme and v) modulating dendritic cell maturation and function, all of which are known mechanisms of action for Tregs (47). As blood samples are relatively limited in pediatric subjects, and many CRIM-positive subjects are now treated pre-emptively with immune-suppressive therapies, validation of the putative Treg epitopes in GAA will require a concerted effort and close collaboration with IOPD families and clinicians.

ADA also develop in IOPD individuals who have splice site mutations that result in indeterminate protein products (6). Fourteen of the 62 subjects were excluded due to presentation with splice site mutations which may not directly change the nGAA sequence (8). As the presence or absence of residual GAA is difficult to assess in these subjects, we have yet to resolve how to accurately predict the tolerance induced by putative Treg epitopes in these subjects. An *in vitro* test (TTBSA) could be developed using their peripheral blood cells that could guide their ADA risk assessment.

No comparison to publicly available tools was made because the type of analysis performed by JanusMatrix is not available elsewhere. These tools have been compared to on-line tools in other settings such as for cancer, please see reference (48). Several additional studies have demonstrated the utility of EpiMatrix and JanusMatrix for identifying HLA DR restricted T effector and putative T regulatory epitopes in human proteins (10, 20). Similar analyses performed by other groups have suggested that ‘self-like’ epitopes may be tolerated or tolerogenic (49).

**Figure 7:**
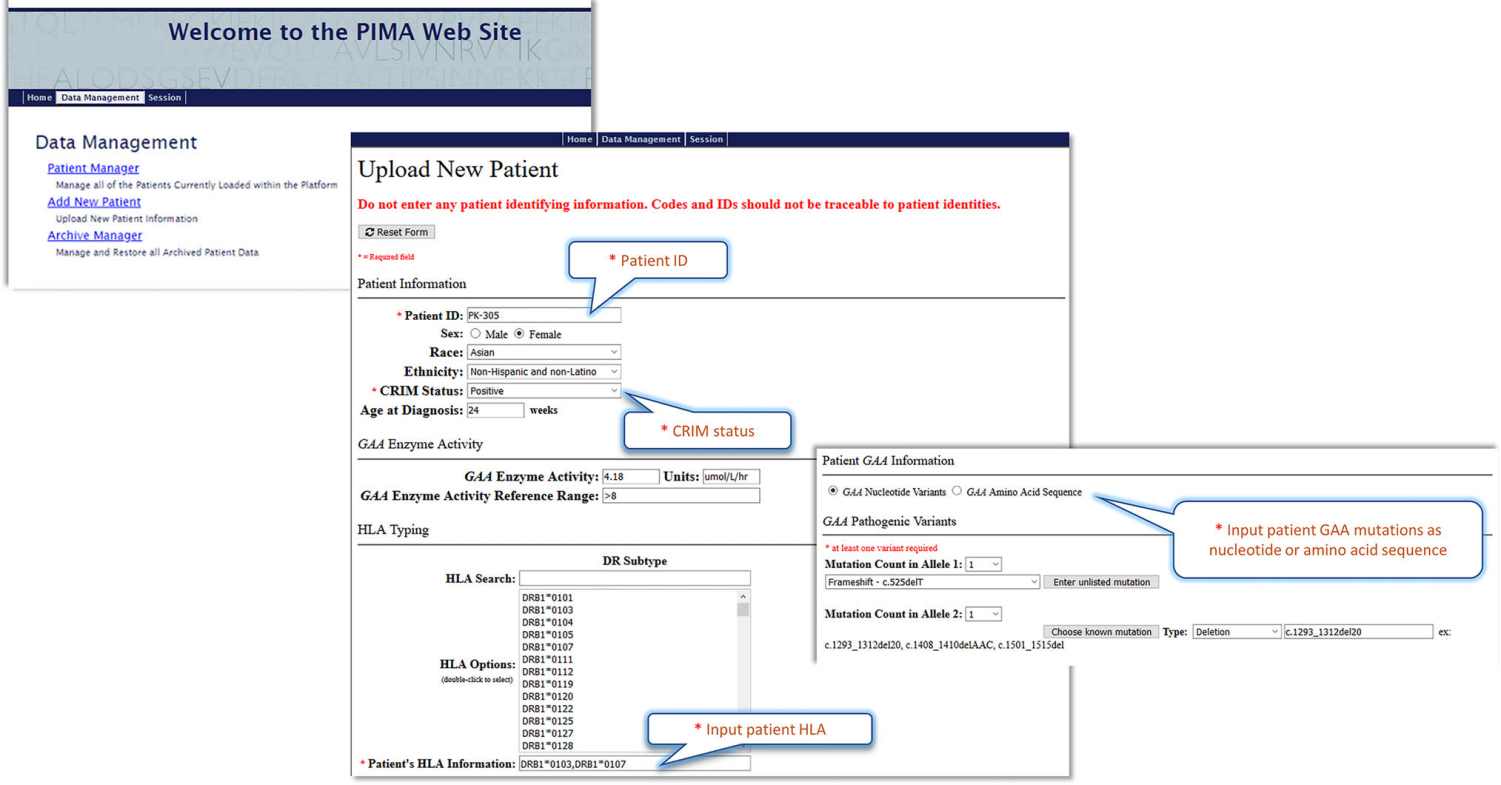
The Pompe PIMA user interface prototype. The upper screenshot shows the Pompe PIMA homepage with data management options. The lower two screenshots from the Upload New Patient page feature the required (*) input fields to generate the IOPD patient’s individualized ADA risk assessment score.

Further validation of these models in prospective studies will be necessary before the models are implemented in clinical settings. The best predictive model (V3J) has been incorporated into a web-based “**P**ersonalized **IM**munogenicity **A**ssessment” tool (PIMA) (**Figure 7**) to facilitate additional research. This website is available for use in pre-clinical research studies. Further validation studies may enable clinicians to input IOPD HLA-type and GAA mutations and generate PIMA scores to predict ADA for their IOPD patients. We anticipate that personalizing treatment using the PIMA tool may assist clinicians in their efforts to improve clinical outcomes for Pompe disease children.

## Data Availability Statement

All relevant data generated for this study are included in the article/**Supplementary Material**. The raw data supporting the conclusions of this manuscript will be made available by the authors to any qualified researcher upon request.

## Ethics Statement

The studies involving human participants were reviewed and approved by Duke Institutional Review Board. Written informed consent to participate in this study was provided by the participants’ legal guardian/next of kin.

## Author Contributions

PSK, ADG, MA, and WDM conceptualized and designed the study, experiments and *in silico* tool development. Experiments, *in silico* implementation of algorithms were performed by MA, WDM, FET, SK, SL, JSY and SM. IOPD patient cohort data was collected by AKD, CL, and PSK. All authors contributed to the article and approved the submitted version.

## Funding

Research reported in this publication was supported by the National Center For Advancing Translational Sciences (NCATS) of the National Institutes of Health under Award Number R43TR002441. The content is solely the responsibility of the authors and does not necessarily represent the official views of the National Institutes of Health. Rhode Island Commerce provided support as part of the Innovate RI Small Business Fund SBIR/STTR Phase I Matching Grant Program, STAC/RI Commerce.

## Conflict of Interest

ADG and WDM are senior officers and shareholders, and SM, FET, SK, MA, and SL are employees of EpiVax, Inc., a company specializing in immunoinformatic analysis. EpiVax, Inc. own patents to technologies utilized by associated authors in the research reported here.

AKD has received grant support from Sanofi Genzyme and the lysosomal disease network. PSK has received research/grant support from Sanofi Genzyme, Valerion Therapeutics, and Amicus Therapeutics. PSK has received consulting fees and honoraria from Sanofi Genzyme, Amicus Therapeutics, Maze Therapeutics, JCR Pharmaceutical and Asklepios Biopharmaceutical, Inc. (AskBio). PSK is a member of the Pompe and Gaucher Disease Registry Advisory Board for Sanofi Genzyme, Amicus Therapeutics, and Baebies. PSK has equity in Asklepios Biopharmaceutical, Inc. (AskBio), which is developing gene therapy for Pompe disease and Maze Therapeutics, which is developing small molecule in Pompe disease.

The remaining authors declare that the research was conducted in the absence of any commercial or financial relationships that could be construed as a potential conflict of interest.

## Abbreviations

Tregitope, Regulatory T Cell epitope; Tregs, Regulatory T Cells; MHC, Major Histocompatibility Complex also referred to Human Leukocyte Antigen (HLA) complex in humans; TCR, T Cell Receptor; IOPD, Infantile Onset Pompe Disease; LOPD, Late-onset Pompe Disease; ERT, Enzyme Replacement Therapy; ITI, Immune Tolerance Induction; ADA, Anti-Therapeutic Antibodies; GAA, acid alpha-glucosidase; rhGAA, recombinant human GAA; nGAA, native or endogenous-expressed GAA of patient; CRIM, cross-reactive immunological material; GSD, glycogen storage disease; HSAT, High and Sustained Antibody Titers SIT, Sustained Intermediate Titers.
