# Supplementary material for: Immune Tolerance-Adjusted Personalized Immunogenicity Prediction for Pompe Disease
Source: Front Immunol. 2021 Jun 16;12:636731. doi: 10.3389/fimmu.2021.636731 (PMC8242953; doi:10.3389/fimmu.2021.636731)

Supplementary Material

Supplementary Table S1. IOPD Cohort and input data used for PIMA risk assessment calculations.

Patient ID	Age at ERT initiation (weeks)	PIMA V1 (Supertype alleles)	PIMA V2, V3, V3J		GAA Mutation(s)	ADA
			Specific HLA DR Allele	HLA DR Allele Proxy, if used		
818-2	21.7	DRB1*0801/ DRB1*1301	DRB1*0801/ DRB1*1301		c.437delT<br>c.2482_2646del	HSAT
818-3	23.5	DRB1*0401/ DRB1*1501	***DRB1*0403/*** DRB1*1501	***DRB1*0404***	c.2482_2646del	HSAT
PK-013	21.7	DRB1*0701/ DRB1*1101	DRB1*0701/ DRB1*1601		c.2236T>C<br>c.2560C>T	HSAT
PK-014	59	DRB1*0901/ DRB1*1101	DRB1*0901/ ***DRB1*1110***	***DRB1*1101***	c.655G>A<br>c.1979G>A	HSAT
PK-015	34.8	DRB1*1101/ DRB1*1301	DRB1*1104/ DRB1*1302		c.2560C>T<br>c.1129G>C<br>c.2770T>C	HSAT
PK-023	20	DRB1*0401/ DRB1*0801	***DRB1*0403/ DRB1*0803***	***DRB1*0404/ DRB1*0801***	c.2238G>A<br>c.1843G>A	SIT
PK-024	13.5	DRB1*0101/ DRB1*0701	DRB1*0101/ DRB1*0701		c.1933G>A	LT
PK-025	30.4	DRB1*1501/ DRB1*1501	***DRB1*1503/ DRB1*1503***	***DRB1*1501/ DRB1*1501***	c.1710C>G<br>c.2560C>T	LT
PK-026	23.5	DRB1*0801/ DRB1*0901	DRB1*0802/ DRB1*0901		c.1802C>T<br>c.1726G>A<br>c.2065G>A<br>c.1099T>C	LT
PK-027	32.2	DRB1*1101/ DRB1*1101	DRB1*1101/ ***DRB1*1602***	***DRB1*1601***	c.2297A>C	LT
PK-029	13.1	DRB1*0801/ DRB1*1301	***DRB1*0811/*** DRB1*1301	***DRB1*0801***	c.1933G>A	LT
PK-030	30.4	DRB1*1501/ DRB1*1501	DRB1*1501/ DRB1*1501		c.655G>A	LT
PK-031	26.1	DRB1*1101/ DRB1*1501	DRB1*1101/ DRB1*1501		c.1210G>A<br>c.2482_2646del	LT
PK-032	28.7	DRB1*0401/ DRB1*0401	***DRB1*0403/ DRB1*0403***	***DRB1*0404/ DRB1*0404***	c.2815_2816del<br>c.1935C>A	LT
PK-033	14.8	DRB1*1101/ DRB1*1501	DRB1*1104/ DRB1*1501		c.2741delinsGAC	LT
PK-034	26.1	DRB1*0401/ DRB1*0801	***DRB1*0406/ DRB1*0803***	***DRB1*0404/ DRB1*0801***	c.1411_1414del<br>c.460_465del	HSAT
PK-035	52.1	DRB1*0701/ DRB1*1301	DRB1*0701/ DRB1*1302		c.1942G>A	HSAT
PK-036	30.4	DRB1*0401/ DRB1*1501	DRB1*0401/ DRB1*1501		c.525delT<br>c.2482_2646del	HSAT
PK-102	9	DRB1*1101/ DRB1*1501	DRB1*1104/ DRB1*1501		c.1293_1312del<br>c.1716C>G	LT
PK-104	4	DRB1*0801/ DRB1*1301	DRB1*0804/ DRB1*1302		c.1082C>T<br>c.2560C>T	LT
PK-105	0	DRB1*0401/ DRB1*1501	DRB1*0401/ DRB1*1502		c.947A>G<br>c.2482_2646del	LT
PK-106	4	DRB1*0101/ DRB1*0301	***DRB1*0102/*** DRB1*0301	***DRB1*0104***	c.2297A>C	LT
PK-107	28	DRB1*0701/ DRB1*1501	DRB1*0701/ DRB1*1502		c.525delT<br>c.1655T>C	LT
PK-109	21	DRB1*0401/ DRB1*1301	DRB1*0405/ DRB1*1302		c.925G>A	HSAT

Patient ID	Age at ERT initiation (weeks)	PIMA V1 (Supertype alleles)	PIMA V2, V3, V3J		GAA Mutation(s)	ADA
			Specific HLA DR Allele	HLA DR Allele Proxy, if used		
PK-110	39	DRB1*0301/<br>DRB1*0401	DRB1*0301/<br>DRB1*0401		c.1799G>T<br>c.525delT	SIT
PK-111	2	DRB1*1101/<br>DRB1*1101	DRB1*1102/<br>DRB1*1104		c.1979G>A<br>c.2560C>T	LT
PK-113	36.5	DRB1*0301/<br>DRB1*0701	DRB1*0301/<br>DRB1*0701		c.525delT<br>c.1841C>A	LT
PK-114	36.5	DRB1*0101/<br>DRB1*1301	DRB1*0101/<br>DRB1*1302		c.1408_1410del<br>c.925G>A	LT
PK-115	31	DRB1*0301/<br>DRB1*1101	DRB1*0301/<br>***DRB1*1103***	***DRB1*1102***	c.1221C>A<br>c.1281G>T<br>c.2296T>A<br>c.1564C>T	LT
PK-116	16	DRB1*0701/<br>DRB1*1301	DRB1*0701/<br>DRB1*1301		c.655G>A<br>c.2167_2179delinsTGCGACGTGG	LT
PK-118	44	DRB1*0401/<br>DRB1*0401	DRB1*0401/<br>DRB1*0405		c.1402A>T<br>c.1841C>A	LT
PK-121	5	DRB1*0401/<br>DRB1*0701	DRB1*0404/<br>DRB1*0701		c.2482_2646del<br>c.670C>T	LT
PK-122	18	DRB1*0401/<br>DRB1*0401	DRB1*0401/<br>DRB1*0401		c.670C>T<br>c.799_803delinsA	SIT
PK-123	25	DRB1*0801/<br>DRB1*1101	DRB1*0804/<br>DRB1*1102		c.525delT<br>c.1979G>A	SIT
PK-016	36.5	DRB1*0401/<br>DRB1*0401	DRB1*0401/<br>DRB1*0404		c.2012T>G<br>c.2188G>T	HSAT
PK-028	2.2	DRB1*0101/<br>Not assigned	DRB1*0101/<br>***DRB1*1401***	***DRB1*13169***	c.525delT<br>c.1642G>T<br>c.1880C>T	LT
PK-101	16	DRB1*0401/<br>DRB1*1501	DRB1*0405/<br>***DRB1*1503***	***DRB1*1501***	c.1710C>G<br>c.953T>C	HSAT
PK-124	23.5	DRB1*0301/<br>DRB1*0401	DRB1*0301/<br>DRB1*0401		c.307T>G<br>c.2482_2646del	HSAT
PK-126	19	DRB1*0101/<br>DRB1*0701	DRB1*0101/<br>DRB1*0701		c.1979G>A<br>c.2560C>T	HSAT
PK-127	19	DRB1*0401/<br>DRB1*0801	***DRB1*0407/***<br>DRB1*0802	***DRB1*0408/***	c.1210G>A	SIT/High
PK-128	42	DRB1*0301/<br>DRB1*1101	DRB1*0301/<br>DRB1*1104		c.706delG<br>c.1805C>T	SIT/Low
PK-125	13	DRB1*1101/<br>DRB1*1301	DRB1*1104/<br>DRB1*1302		c.307T>G<br>c.917C>T<br>c.2528T>C	LT
PK-120	20.9	DRB1*1101/<br>DRB1*1501	DRB1*1101/<br>DRB1*1501		c.148_859-11del<br>c.685_686insCGGC<br>c.1726G>A	HSAT
PK-130	7	DRB1*0101/<br>DRB1*0301	DRB1*0103/<br>DRB1*0301		c.953T>C<br>c.2482_2646del	LT
PK-131	8	DRB1*0101/<br>DRB1*0301	DRB1*0101/<br>DRB1*0301		c.925G>A<br>c.1841C>A	LT
PK-134	6.7	DRB1*0801/<br>DRB1*0801	DRB1*0802/<br>DRB1*0802		c.2104C>T	LT
PK-136	12	DRB1*0301/<br>DRB1*1501	DRB1*0301/<br>DRB1*1501		c.1441T>C<br>c.2481+102_2646+31del	LT
PK-144	1.1	DRB1*0901/<br>DRB1*1501	DRB1*0901/<br>DRB1*1502		c.1935C>A<br>c.1726G>A<br>c.2024_2026del	LT

Bolded and italicized allele is for a selected proxy allele based on pocket similarity.

Supplementary Table S2. Selection of putative regulatory and T effector epitopes in GAA. The human GAA complete sequence was evaluated for potential regulatory epitopes using the EpiMatrix and Janus Matrix tools. Table shows the EpiMatrix cluster score (binding probability Z-score) and the number of human matches to the other human proteins that are conserved within the GAA-epitope at its TCR-facing residues. JanusMatrix score is a measure of TCR conservation where a value > 2 is considered significant and may be putative tolerogenic epitopes. Peptides were synthesized and evaluated in the *in vitro* HLA binding assay and for their inhibitory capacity in the Tetanus Toxoid Bystander Suppression assay (TTBSA). Peptides hGAA 1a, 1b, 6 and 11 (denoted by * in the table) were shown to exhibit significant suppression in the TTBSA similar to well-established Tregitopes. Additionally, two GAA-derived T effector epitopes having predicted binding across multiple HLA alleles and low cross-conservation (JMX scores <2) were selected and included in the TTBSA as negative controls.

Peptide Name	EpiMatrix cluster score	Number of human matches	Janus Homology score
hGAA-1a *	24.34	41	4.75
hGAA-1b *	32.32	29	4.50
hGAA-2	17.84	33	4.50
hGAA-3	17.80	14	3.64
hGAA-4	6.54	14	3.20
hGAA-5	38.19	29	4.05
hGAA-6 *	40.21	21	5.30
hGAA-7	22.09	28	4.83
hGAA-8	30.76	37	7.29
hGAA-9	51.13	27	3.32
hGAA-10	12.88	24	7.29
hGAA-11 *	22.32	22	2.67
hGAA-12	0.96	7	3.50
hGAA-13	1.01	7	7.00
hGAA-14	2.06	18	9.00
hGAA-15	5.60	7	2.25
hGAA-16	12.08	12	2.40
hGAA-17	2.65	10	4.00
hGAA-18	0.74	8	8.00
hGAA-19	11.26	10	3.33
hGAA-20	16.47	13	2.43
hGAA-Teff-1	5.3	8	1.67
hGAA-Teff-2	18.94	6	1.44

Supplementary Figure S1. Comparison of the area under the ROC curve (AUC) among the univariate and multivariable logistic regression models. A) AUC values to quantify model accuracy ranged from 0.58 to 0.67 for the univariate logistic regression model, which incorporates PIMA V1-V3J scores. **B**) The AUC values ranged from 0.74 to 0.76 for the multivariable logistic regression model, which incorporates both PIMA V1-V3J scores and age in weeks at initiation of ERT. While accuracy using PIMA scores alone was moderate, the AUCs for the four scoring algorithms were increased above 0.7 after incorporating the age variable, improving the model's ability to classify the ADA status of the Pompe patients. AUCs were calculated using the pROC package with R. Notably, while the addition of age increased area under the ROC curve for models employing all PIMA versions, due to the fixed age values across models and small sample size, it also reduced sensitivity, specificity, PPV and NPV.

A)

Model 1	AUC	PPV	NPV	Sens	Spec
PIMA V1	0.60	48%	71%	68%	52%
PIMA V2	0.59	47%	72%	74%	45%
PIMA V3	0.58	46%	70%	68%	48%
PIMA V3J	0.67	57%	76%	68%	66%

B)

Model 2	AUC	PPV	NPV	Sens	Spec
PIMA V1 + Age/ERT	0.75	36%	53%	63%	28%
PIMA V2 + Age/ERT	0.74	36%	53%	63%	28%
PIMA V3 + Age/ERT	0.74	36%	53%	63%	28%
PIMA V3J + Age/ERT	0.76	41%	63%	63%	41%

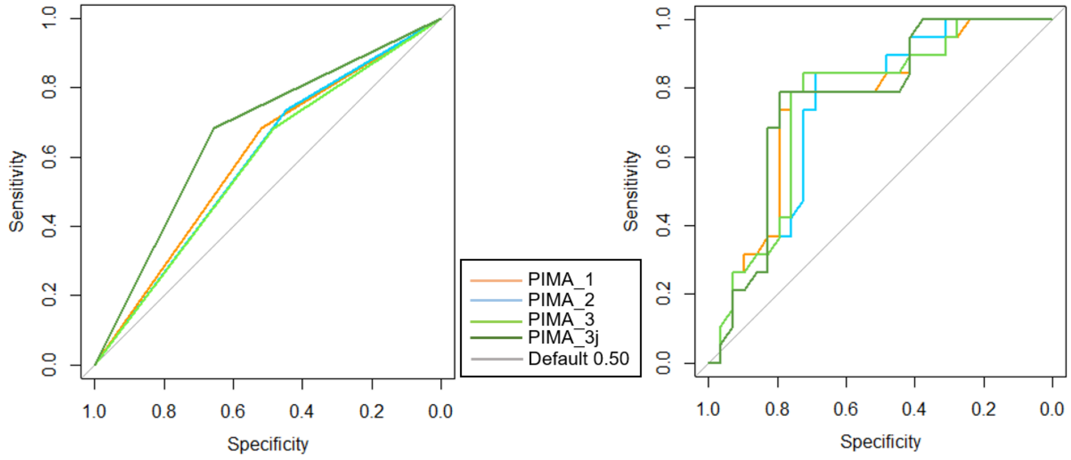

Supplementary Figure S2. GAA-derived T effector peptides did not inhibit memory CD4 T cell response to Tetanus Toxoid (TT) in healthy donors. PBMCs from healthy donors were stimulated with 0.5 µg/ml of TT with or without FV621 or GAA-derived Teff peptides and analyzed at six days post-stimulation by flow cytometry for inhibition of CD4+ T cell proliferation. Data show the CD4 T cell proliferation averaged from 3 healthy donors in the experiments. FV621 was used as a positive control where significant suppressive capacity of TT-induced CD4+ T cell proliferation was observed. P values **** = <0.0001 represents statistical significance between TT stimulation alone vs TT+ peptide using a two-tailed t test.

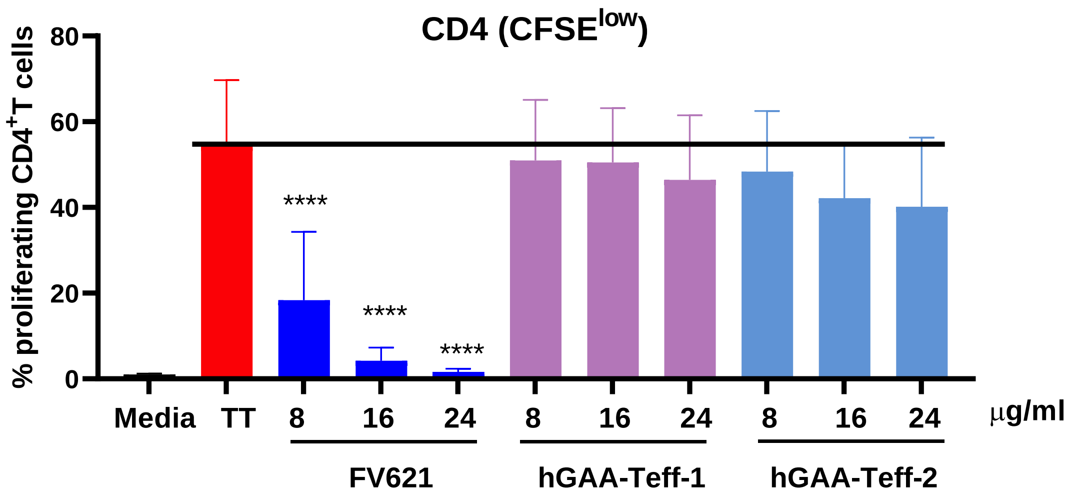

Supplement: Supplementary file 1 [file DataSheet_1.pdf]
